# Oral microbiome diversity shapes the association between sleep duration and depression

**DOI:** 10.3389/fneur.2024.1442557

**Published:** 2024-09-13

**Authors:** Yan Liu, Ling Zhang, Can Yang, Liping Zhi, Xu Steven Xu, Min Yuan

**Affiliations:** ^1^Department of Health Data Science, Anhui Medical University, Hefei, Anhui, China; ^2^Clinical Pharmacology and Quantitative Science, Genmab Inc., Princeton, NJ, United States; ^3^MOE Key Laboratory of Population Health Across Life Cycle, Hefei, Anhui, China

**Keywords:** sleep duration, oral microbiome diversity, depression, interaction, National Health and Nutrition Examination Survey

## Abstract

**Background:**

Emerging research suggests the relationship between the oral microbiome and sleep duration with depression, however, the precise mechanisms by which oral microbial diversity influences the sleep-depression nexus remain to be elucidated.

**Methods:**

We analyzed data from 4,692 participants in the National Health and Nutrition Examination Survey (NHANES), incorporating key demographic variables, oral microbiome diversity metrics, sleep duration, and depression assessment variables. Classical multidimensional scaling facilitated dimensionality reduction, while unsupervised clustering divided participants into groups based on β-diversity dissimilarity matrices. We examined the moderating effects of oral microbiome diversity on the sleep-depression relationship by incorporating interaction terms sleep-oral microbiome diversity into multiple linear regression models.

**Results:**

Our analysis revealed a U-shaped relationship between sleep duration and depression. Specifically, α-diversity was a significant moderator, with reduced diversity linked to an increased depression risk in participants with insufficient sleep. Regarding β-diversity, using both Bray-Curtis and UniFrac distance measures, Cluster 2 exhibited the strongest associations in sleep-deprived individuals (Bray-Curtis: *β* = 1.02, *p* < 0.001; Weighted UniFrac: *β* = 0.91, *p* < 0.001). In contrast, Cluster 1 displayed notable effects in individuals with excessive sleep (Bray-Curtis: *β* = 0.63, *p* = 0.008). Additionally, Cluster 3 was prominently associated with depression in sleep-deprived participants using unweighted UniFrac distance (*β* = 0.93, *p* < 0.001), and Cluster 2 was significant among those with excessive sleep across both unweighted (*β* = 0.80, *p* = 0.0004) and weighted UniFrac distances (*β* = 0.60, *p* = 0.001).

**Conclusion:**

This study highlights the crucial role of oral microbiome diversity in moderating the U-shaped relationship between sleep duration and depression risk.

## Introduction

Depression is a widespread and complex mental health disorder, affecting over 300 million people globally and ranking as a major contributor to the global burden of disease according to the World Health Organization (1, 2). The etiology of depression is multifaceted, involving genetic, biological, environmental, and psychological factors (3–6). Recent advances in microbiome research, particularly in the gut, have underscored their profound influence on mental health (7–11). This has sparked a burgeoning interest in the oral microbiome, another critical component of the microbial ecosystem, and its potential impact on mental health disorders.

The oral microbiome consists of a diverse community of microorganisms such as bacteria, fungi, viruses, and archaea, situated within the oral cavity (12, 13). Boasting over 700 bacterial species, the oral cavity ranks as one of the most diverse and densely populated microbiota in the human body, second only to the gut. This diversity establishes the oral cavity as the most bacterially rich region in the human body (14). These microorganisms play vital roles in maintaining oral health, including preventing pathogen colonization, facilitating digestion, and modulating immune responses. Moreover, the oral cavity’s significant vascularity and frequent exposure to bacteremia from microbial migration across mucosal linings position it as a pivotal area where the microbiome can influence a variety of systemic diseases. These include cardiovascular disease, diabetes, rheumatoid arthritis, and notably, mental health disorders such as depression (15–23).

Emerging studies have unveiled fascinating connections between the oral microbiome and mental health disorders (21, 24). Changes in the composition of the oral microbiome, for instance, have been associated with conditions like depression and anxiety. Potential mechanisms involve the gut-brain axis, where microbial metabolites and immune responses originating in the oral cavity may impact neural circuits and mood regulation, thereby affecting mental health (16, 25). These insights illuminate the intricate interplay within the microbiome and open new paths for understanding and potentially addressing mental health disorders.

Two principal dimensions of microbial diversity, that is α-diversity and β-diversity, are used to describe the complexity of oral microbiome (26). α-diversity, measured by metrics such as OTU richness, Faith’s phylogenetic diversity, Shannon-Wiener index, and inverse Simpson index, provides insights into the variety and abundance of species within a specific environment. Conversely, β-diversity, assessed through metrics like unweighted and weighted UniFrac dissimilarity and Bray-Curtis distance, examines the differences in microbial compositions across various environments, helping identify unique microbial signatures.

Sleep is fundamental for maintaining physical health, emotional well-being, and cognitive performance, profoundly influencing overall quality of life (27–29). Adequate sleep is crucial for bodily healing and repair, immune function, and hormone regulation, which affect appetite and stress levels. It bolsters mental health by enhancing mood, reducing anxiety, and bolstering resilience against stress. From a cognitive standpoint, sufficient sleep is essential for sharpening attention, enhancing learning and memory, and improving decision-making skills. Therefore, ensuring good quality sleep is vital for well-being and life satisfaction, involving practices such as adhering to a regular sleep schedule, creating a conducive sleep environment, and managing pre-sleep routines to encourage restfulness. Moreover, research consistently shows a robust connection between sleep patterns and mental health issues, especially depression (30, 31). Disruptions in sleep, including insomnia and hypersomnia, are strongly linked to a higher risk of depression onset. A U-shaped relationship between sleep duration and depression has been identified, indicating that both short and excessively long sleep durations are associated with higher depression rates compared to moderate sleep durations (32). This relationship suggests an optimal sleep range that supports mental health, while deviations from this range, either insufficient or excessive sleep, may contribute to the onset and severity of depressive symptoms (33). Ideally, adults should aim for 7–9 h of quality sleep per night to support overall well-being (34).

Although existing studies have associated sleep and oral microbiome diversity to depression, the relation between sleep, the oral microbiome, and depression remains complex and not yet fully understood. This research aims to uncover how variations in the oral microbiome could potentially moderate the relation between sleep duration and depression in the U.S. population, offering new perspectives on how microbial diversity can affect mental health outcomes.

## Methods

### Study sample

This study utilized data from National Health and Nutrition Examination Survey (NHANES) collected between 2009 and 2012. Detailed information on the survey objective, target population, and data collection methods can be found at: https://wwwn.cdc.gov/nchs/nhanes/. Briefly, participants under 20 years old were excluded from the current study. Only participants with complete demographic characteristics (age, sex, race, education, marital status, poverty-income ratio) and lifestyle variables (smoking, self-reported sleep duration, body mass index, dietary inflammatory index, and leisure-time physical activity time) and oral microbial diversity data were included in this study. After eliminating samples with missing data, the dataset included 4,692 samples for analysis. Data from 2009 to 2012 were chosen due to the availability of oral microbiome diversity information during those years. No institutional review board approval was required as the data is publicly accessible on the NHANES official website.

### Covariates

We considered several covariates associated with oral microbiome and depression. Demographic factors included age (20–39, 40–59, >60 years), gender, race/ethnicity (white, black, Mexican American, others), education (below high school, high school, college or above), marital status (married, living alone), and poverty income ratio (PIR). Lifestyle factors comprised smoking status (nonsmokers, smokers), self-reported sleep duration (hours per day, hrs/day), sedentary time (minutes/day, min/day), and body mass index categories (below 25, 25–30, 30–35, above 35 kg/m^2^). We assessed dietary habits using the Dietary Inflammatory Index (DII) (35) and leisure time physical activity based on recent exercise and active hobbies.

### Exposures

In this study, we focused on the duration of sleep as the key exposure variable. Sleep duration data for all participants were obtained from the NHANES dataset for the years 2009–2012. For individuals aged 20 and above, we categorized their sleep hours into four groups: extra lack sleep (<=4 h), insufficient sleep (5–7 h), adequate sleep (7–9 h), and excessive sleep (> = 10 h), in accordance with sleep duration guidelines recommended by the US Centers for Disease Control and Prevention (CDC) (36).

### Outcomes

The primary outcome in this study was depression, assessed using the Patient Health Questionnaire-9 (PHQ-9), a self-assessment tool that measures the severity of depressive symptoms. The PHQ-9 comprises nine questions related to common depression symptoms, such as low mood, loss of interest, sleep changes, fatigue, appetite changes, feelings of guilt, difficulty concentrating, psychomotor changes, and thoughts of self-harm or suicide. Each question is scored from 0 to 3, with 0 indicating the absence of the symptom and 3 indicating its presence nearly every day. The total score, ranging from 0 to 27, reflects the severity of depression, with higher scores indicating greater severity. Clinically, scores of 0–4 are considered normal, while scores above 4 indicate depression, categorized as mild (5–9), moderate (10–14), and severe (15–27) (37).

### Moderators

In this study, we examined the role of oral microbiome diversity as a moderator. Oral rinse samples were collected using mouthwash for 5 s, followed by three 5-s gargles. DNA extraction was performed using a Puregene DNA purification kit. α-diversity and β-diversity were calculated from the raw sequence data. Details for deriving α-diversity and β-diversity were described elsewhere (26).

α-diversity metrics (OTU richness, Faith’s phylogenetic diversity, Shannon-Wiener index, and inverse Simpson index) were used to assess microbial diversity within individual samples. These metrics were calculated with rarefaction values of 10,000, capturing both richness and evenness within microbial communities. β-diversity metrics (unweighted UniFrac, weighted UniFrac, and Bray–Curtis dissimilarity) measured dissimilarity between individuals’ microbiome compositions (38).

### Statistical analysis

Sample characteristics were summarized using median (IQR) for continuous variables and counts (%) for categorical variables. *p*-values for the initial comparison between characteristic variables and depression stages were calculated using the Kruskal-Wallis rank sum test for continuous data and Pearson’s Chi-squared test for categorical data.

We applied a multiple linear regression model to assess the relationship between depression scores and α-diversity metrics while accounting for demographic and lifestyle variables. The fourth quartile, representing high diversity, served as the reference group. For β-diversity metrics, we employed classic multidimensional scaling (MDS) for dimensionality reduction, followed by K-means clustering to assign participants to clusters. The optimal number of clusters for each β-diversity metric was determined using the silhouette measure and elbow plot. Multiple linear models were then used to explore the association between clustering labels and depression scores, with adjustments for potential covariates.

**Figure 1:**
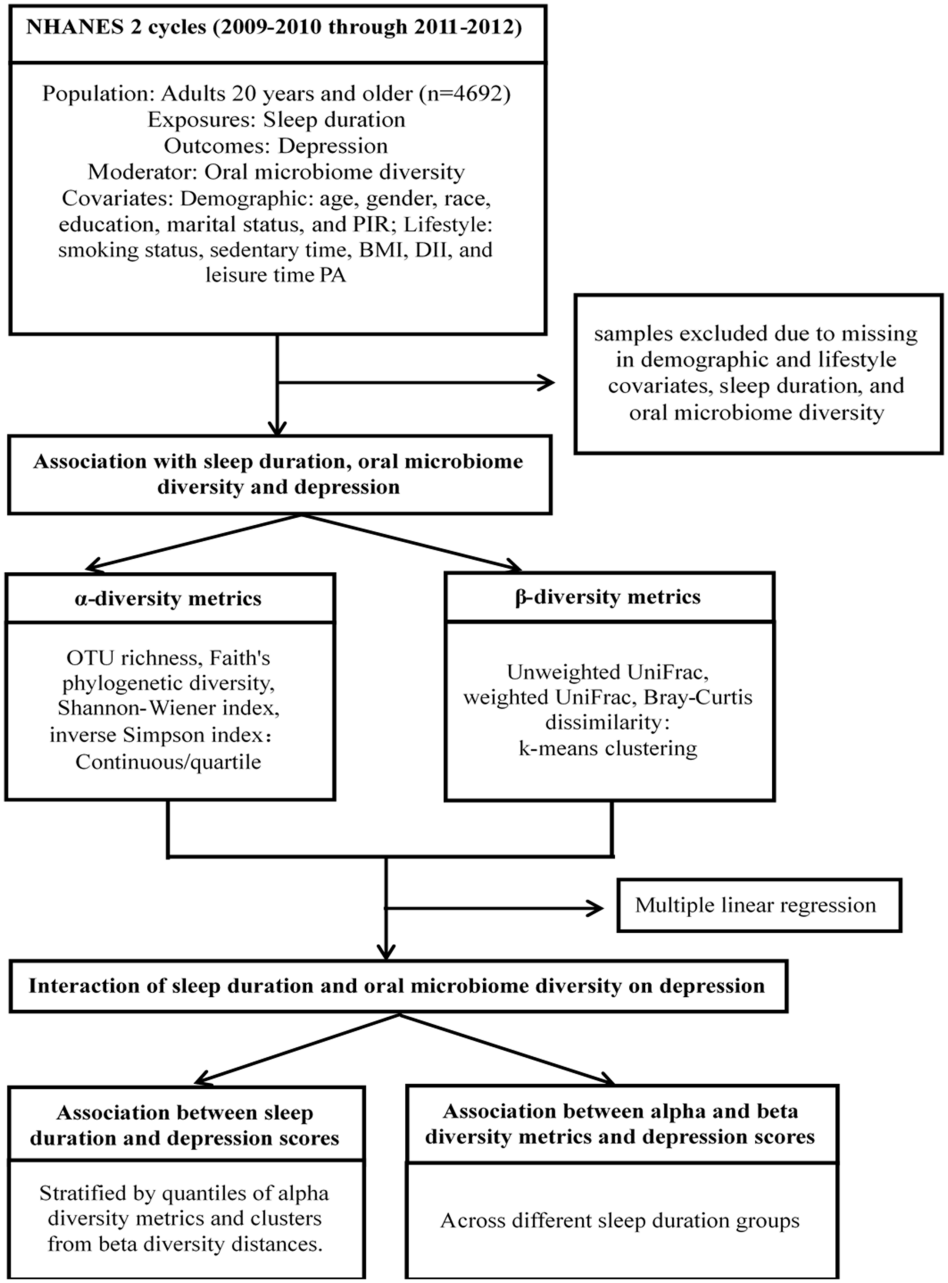
Schematic diagram of data preprocessing and analytic methods.

To explore the role of oral microbiome diversity as a moderator, we introduced multiplicative interaction terms (sleep hour category × α-diversity metrics or clustering membership based on β-diversity dissimilarity matrix). The interaction effects were quantified as the conditional effects of sleep duration on depression for α-diversity quarters and β-diversity clusters. The significance threshold was set at 0.05. Figure 1 outlines our data processing and statistical analysis workflow. All analyses were performed using R software, version 4.0.2.

## Results

The mean age of the participants is 48.9 years with a standard deviation of 11.3. Roughly, half of the participants fell within the 40–60 age group, with 51% being male (Table 1). Across all participants, the average OTU richness, Faith’s phylogenetic diversity, Shannon-Wiener index, and inverse Simpson index were 129 (SD 44), 14.5 (SD 3.5), 4.60 (SD 0.71), and 0.90 (SD 0.07), respectively. These measures of α-diversity exhibited a consistent decrease with increasing depression stages. The differences between the two alpha-diversity metrics (Shannon-Wiener index and inverse Simpson index) and depression stage, though relatively small, reached statistical significance at the 0.05 level (*p* = 0.002 and 0.012). The mean PHQ-9 depression score was 3.4 (SD 4.6). Participants in the severe depression stage exhibited the lowest PIR, leisure time physical activity duration, and sleep duration, while having the highest diet inflammatory index.

**Table 1:** Basic characteristic of the NHANES (2009–2012) 4692 analytical samples by different stage of depression.

Characteristic	Total (*N* = 4,692)	Normal (*N* = 3,470)	Depression stage			
			Mild (*N* = 735)	Moderate (*N* = 290)	Severe (*N* = 197)	*p*
Age (year)						0.003
20–39	1,208 (26%)	898 (26%)	199 (27%)	71 (24%)	40 (20%)	
40–59	2,351 (50%)	1,696 (49%)	372 (51%)	160 (55%)	123 (62%)	
Above 60	1,133 (24%)	876 (25%)	164 (22%)	59 (20%)	34 (17%)	
Gender						<0.001
Male	2,385 (51%)	1,883 (54%)	328 (45%)	110 (38%)	64 (32%)	
Female	2,307 (49%)	1,587 (46%)	407 (55%)	180 (62%)	133 (68%)	
Race/Ethnicity						0.4
White	738 (16%)	545 (16%)	118 (16%)	47 (16%)	28 (14%)	
Black	468 (10%)	329 (9.5%)	76 (10%)	39 (13%)	24 (12%)	
Mexican American	1,091 (23%)	799 (23%)	182 (25%)	61 (21%)	49 (25%)	
Other	2,395 (51%)	1,797 (52%)	359 (49%)	143 (49%)	96 (49%)	
Education						<0.001
<High School	1,111 (24%)	737 (21%)	201 (27%)	96 (33%)	77 (39%)	
High School	2,337 (50%)	1,693 (49%)	391 (53%)	153 (53%)	100 (51%)	
College or Above	1,244 (27%)	1,040 (30%)	143 (19%)	41 (14%)	20 (10%)	
Marriage						<0.001
Married	3,045 (65%)	2,411 (69%)	415 (56%)	134 (46%)	85 (43%)	
Live alone	1,647 (35%)	1,059 (31%)	320 (44%)	156 (54%)	112 (57%)	
Smoking Status						
Never	2,516 (54%)	1,973 (57%)	348 (47%)	115 (40%)	80 (41%)	<0.001
Former	1,103 (24%)	842 (24%)	160 (22%)	57 (20%)	44 (22%)	
Current	1,073 (23%)	655 (19%)	227 (31%)	118 (41%)	73 (37%)	
BMI (kg/m^2^)						<0.001
Below 25	1,159 (25%)	884 (25%)	159 (22%)	77 (27%)	39 (20%)	
25–29.9	1,574 (34%)	1,246 (36%)	220 (30%)	65 (22%)	43 (22%)	
30–34.9	1,071 (23%)	770 (22%)	166 (23%)	79 (27%)	56 (28%)	
Above 35	888 (19%)	570 (16%)	190 (26%)	69 (24%)	59 (30%)	
PIR	2.63 (1.62)	2.86 (1.67)	2.27 (1.60)	1.68 (1.40)	1.37 (1.21)	<0.001
Sleep time (hrs/day)	6.72 (1.36)	6.85 (1.26)	6.49 (1.49)	6.26 (1.83)	5.97 (1.90)	<0.001
Sedentary time (min/day)	340 (201)	337 (199)	352 (202)	338 (210)	361 (212)	0.12
Leisure-time PA (min/day)	36 (100)	37 (105)	33 (88)	36 (87)	25 (71)	0.2
Dietary inflammatory index	1.40 (1.81)	1.26 (1.84)	1.67 (1.78)	1.96 (1.63)	2.10 (1.75)	<0.001
OTU richness	130 (44)	130 (43)	130 (46)	129 (47)	120 (48)	0.10
Faith’s phylogenetic diversity	14.5 (3.5)	14.5 (3.4)	14.6 (3.5)	14.5 (3.7)	13.7 (3.9)	0.2
Shannon-Weiner index	4.60 (0.70)	4.62 (0.68)	4.58 (0.76)	4.60 (0.73)	4.38 (0.83)	0.002
Inverse Simpson index	0.90 (0.06)	0.90 (0.06)	0.90 (0.07)	0.90 (0.05)	0.89 (0.07)	0.011
Depression scores	3.3 (1.4)	1.1 (1.3)	6.6 (1.3)	11.6 (1.4)	18.3 (3.1)	<0.001

### Association with sleep duration, oral microbiome diversity, and depression

After adjusting for covariates, we observed significant differences in depression scores between the extra lack, insufficient sleep, and excessive sleep groups compared to the sufficient sleep duration group. An interesting U-shaped relationship emerged, signifying that individuals with either too little or too much sleep during the night had higher depression scores compared to those with sufficient sleep duration (beta = 0.65 [0.52, 0.69] and 0.35 [0.20, 0.51], all *p* values<0.001). Notably, the regression coefficient for the extra lack sleep group was larger than that for the excessive sleep group, suggesting that insufficient sleep posed a greater risk for depression than excessive sleep.

We employed multiple linear regression models to assess the relationship between depression scores and four α-diversity metrics, both as continuous variables and divided into quarters (Table 2). All four α-diversity metrics showed significant associations with depression scores after adjusting for covariates. The negative associations indicated that individuals with higher α-diversity experienced lower depression scores. Notably, the lowest quartile (Q1) of α-diversity had the more pronounced effect on depression compared to higher quartiles (Q2-Q4), suggesting a nonlinear relationship between the diversity and depression scores and the relationship potential leveled off at the high diversity.

**Table 2:** Association results of four alpha-diversity metrics, three beta-diversity metrics and depression using the multiple regression models adjusting for possible confounders.

		Beta	95% CI	*p*-value
Sleep duration	Sufficient (7-9 h)	—	—	
	Extra lack (1-4 h)	0.61	0.52, 0.69	<0.001
	Insufficient (5-7 h)	0.15	0.10, 0.19	<0.001
	Excessive (> = 10 h)	0.35	0.20, 0.51	<0.001
OTUs	Q4	—	—	
	Q3	0.05	−0.01, 0.11	0.13
	Q2	0.05	−0.01, 0.11	0.093
	Q1	0.11	0.05, 0.17	<0.001
OTUs (continuous)		−0.13	−0.18, −0.07	<0.001
FaPhyloDiv	Q4	—	—	
	Q3	0.04	−0.02, 0.10	0.2
	Q2	0.06	0.00, 0.12	0.052
	Q1	0.11	0.05, 0.17	<0.001
FaPhyloDiv (continuous)		−0.01	−0.02, −0.01	<0.001
InvSimpson	Q4	—	—	
	Q3	0.01	−0.05, 0.07	0.7
	Q2	0.03	−0.03, 0.09	0.4
	Q1	0.10	0.04, 0.16	<0.001
InvSimpson (continuous)		−0.46	−0.79, −0.13	0.007
ShanWienDiv	Q4	—	—	
	Q3	−0.01	−0.07, 0.05	0.9
	Q2	0.04	−0.02, 0.11	0.15
	Q1	0.11	0.04, 0.17	<0.001
ShanWienDiv (continuous)		−0.07	−0.10, −0.04	<0.001
Braycurtis distance	Cluster 1	—	—	
	Cluster 2	0.09	−0.33, 0.52	0.7
	Cluster 3	−0.02	−0.42, 0.38	>0.9
	Cluster 4	0.13	−0.28, 0.54	0.5
Unweighed Unifrac	Cluster 1	—	—	
	Cluster 2	0.30	−0.10, 0.70	0.14
	Cluster 3	0.17	−0.17, 0.51	0.3
	Cluster 4	0.02	−0.29, 0.32	>0.9
Weighed Unifrac	Cluster 1	—	—	
	Cluster 2	0.05	−0.31, 0.40	0.8
	Cluster 3	−0.12	−0.45, 0.20	0.5
	Cluster 4	0.45	0.09, 0.80	0.013

In this study, low-dimensional β-diversity distances were separately clustered resulting in the classification of samples into four classes based on BrayCurtis, unweighted, and weighted Unifrac distances, as determined by the silhouette criterion. The distribution of samples in each cluster was provided in Supplementary Figure S1. We then analyzed the associations between these clustering results and depression scores (Table 2). Notably, we found that only Cluster 4, determined by the weighted Unifrac distance, exhibited a significant difference when compared to Cluster 1 (beta = 0.45 [0.09, 0.80], *p* = 0.013). There were no significant findings for the BrayCurtis and unweighted Unifrac distances.

### Moderator effect of oral microbiome diversity on sleep duration and depression

**Figure 2:**
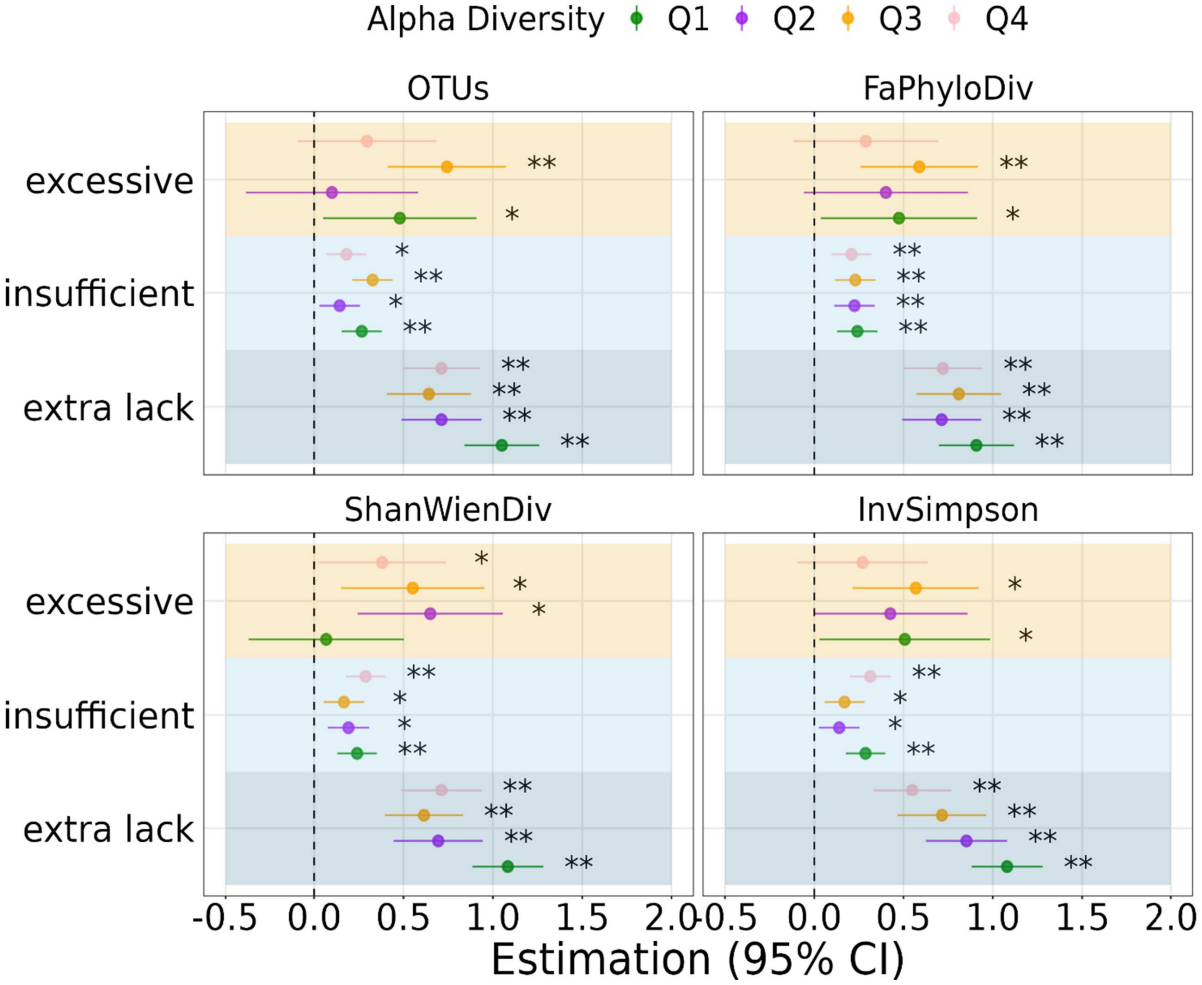
Regression coefficient of the association between sleep duration and depression stratified by quantiles of α-diversity. Significant level: 0.05 (*) and 0.01 (**).

**Figure 3:**
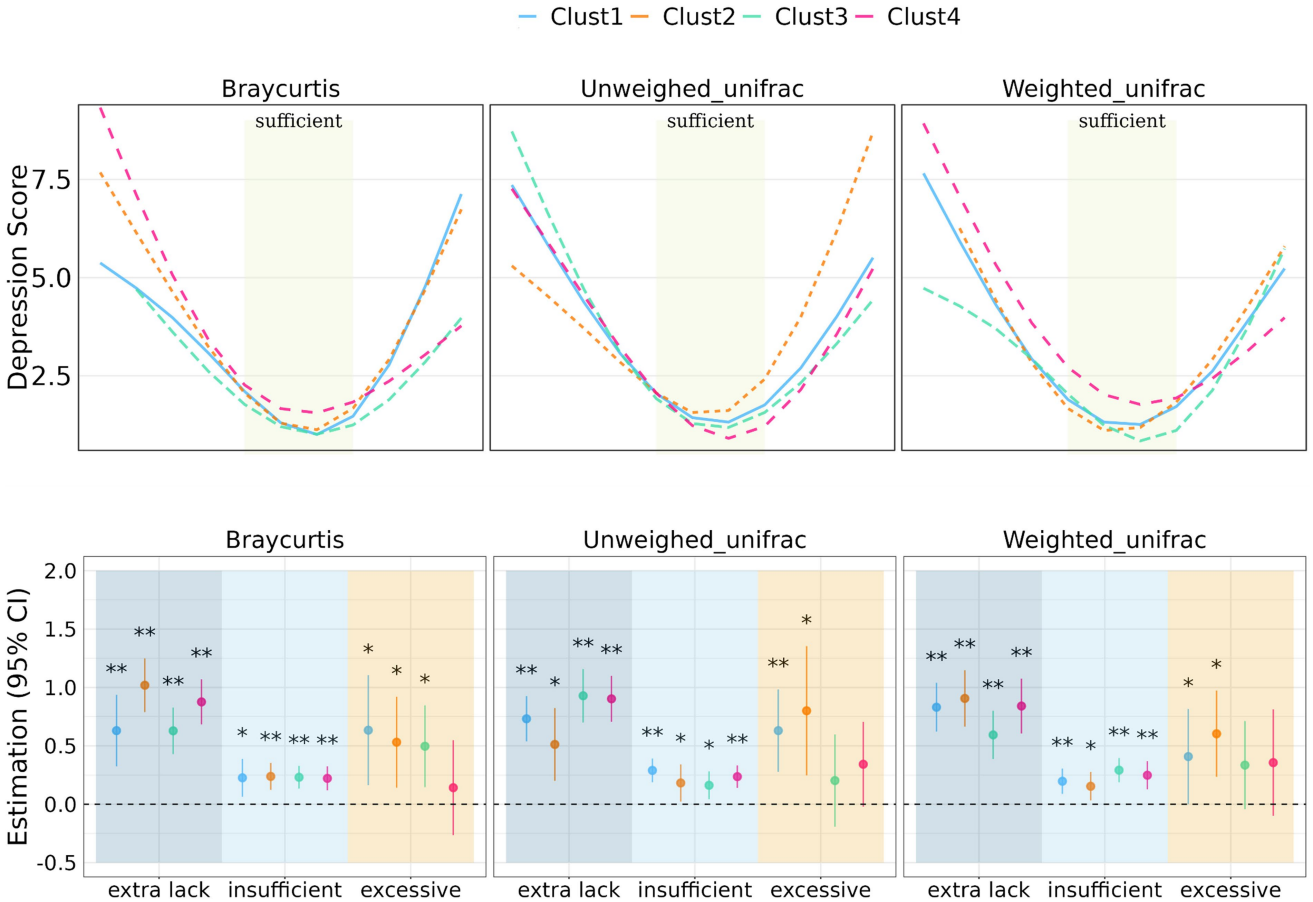
Prediction of depression score with sleep duration stratified by clusters of β-diversity metrics. Regression coefficient of the association between sleep duration and depression score stratified by beta-diversity clusters. Significant level: 0.05 (*) and 0.01 (**).

Figures 2, 3B depict the relationship between sleep duration and depression scores, stratified by quantiles of α-diversity metrics and clusters from β-diversity distances. α-diversity was found to moderate the association between sleep duration and depression, with individuals in the low α-diversity groups within the lack of sleep category experiencing more pronounced effects on depression. The regression coefficients were 1.05, 0.91, 1.08, and 1.08 for the lower OTU-lack sleep, low Faith’s phylogenetic diversity-lack sleep, low Shannon-Wiener diversity-lack sleep, and low Inverse Simpson-lack sleep groups, respectively.

In terms of β-diversity measured by BrayCurtis distance, Cluster 2 had the most significant effects among individuals with extra sleep lack (beta = 1.02 [0.79, 1.25], *p* < 0.001), while Cluster 1 exhibited the most significant effects among individuals with excessive sleep duration (beta = 0.63 [0.16, 1.01], *p* = 0.008). For unweighted UniFrac distance-measured beta-diversity, Cluster 3 had the most significant effects among individuals lacking sleep (beta = 0.93 [0.70, 1.16], *p* < 0.001), and Cluster 2 had the most significant effects among individuals with excessive sleep duration (beta = 0.80 [0.25, 1.35], *p* = 0.0004). When considering weighted UniFrac distance, Cluster 2 had the most significant effects among individuals lacking sleep (beta = 0.91 [0.66, 1.15], *p* < 0.001), and excessive sleep duration (beta = 0.60 [0.23, 0.97], *p* = 0.001).

**Figure 4:**
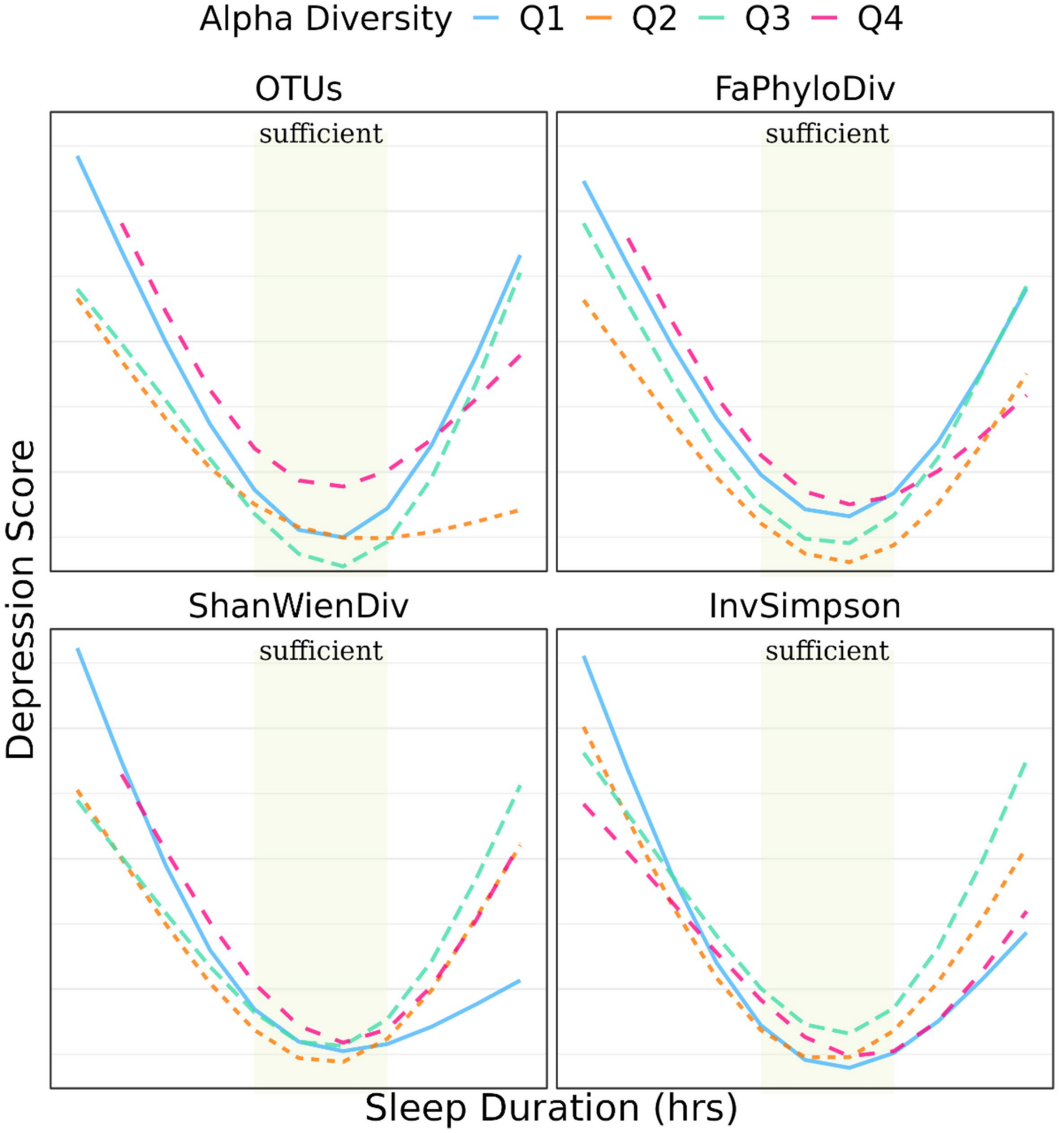
Prediction of depression score with sleep duration stratified by quantiles of α-diversity metrics.

Figures 3A, 4 illustrate the relationship between α-and β-diversity metrics and depression scores across different sleep duration groups. Figure 4 demonstrates the distribution of depression scores among various sleep duration groups, categorized by quantiles of α-diversity. Notably, depression scores exhibited distinct patterns across α-diversity within each sleep duration group. For instance, individuals with extra short sleep hours tended to have higher depression scores when α-diversity (ShanWienDiv and InvSimpson) was relatively low; whereas those with excessive sleep hours showed higher depression scores when α-diversity was richer.

Regarding the distribution of depression scores within different BrayCurtis diversity clusters, individuals in the extra sleep lack group had considerably higher depression scores in Cluster 2 and 4 compared to Cluster 1 and Cluster 3, while in the excessive sleep group, Clusters 1 and 2 had higher depression scores than Cluster 3 and 4. In the case of unweighted UniFrac diversity clusters, individuals in the extra sleep lack group had notably higher depression scores in Cluster 1, 3 and 4 compared to Cluster 2, whereas the excessive sleep group exhibited the highest depression scores in Cluster 2 and the lowest in Cluster 3. Additionally, the distribution of depression scores among individuals in different weighted UniFrac diversity clusters was relatively similar.

### Sensitivity analysis

We assessed the robustness of our findings through three aspects. Firstly, we conducted analyses while controlling for various covariates to ascertain the stability of the results. Secondly, we employed different clustering methods for β-diversity. Finally, we utilized different seeds during the clustering process. All of these approaches yielded consistent results, underlining the robustness of our findings.

## Discussion

Significant association between sleep and depression has raised considerable attention in the field of public health. Disrupted sleep can lead to imbalances in mood-regulating neurotransmitters like serotonin and dopamine, elevate inflammatory markers, and enhance the body’s stress response by increasing cortisol levels (27–30, 39–42). Numerous studies highlight a bidirectional relationship between sleep duration and depression (43). In our analysis, we observed a U-shaped relationship between sleep duration and depression, consistent with existing literature that suggests both short and long sleep durations are associated with an increased likelihood of experiencing depressive symptoms.

Changes in the oral microbiome have been associated with mental health issues such as depression (21, 24). For instance, Li et al. found a genetic link between the oral microbiome and depression and anxiety using genome-wide association study (GWAS) summary data, by examining polygenic risk scores (PRSs) of 285 salivary microbiomes and 309 tongue dorsum microbiomes (32). Similarly, Wingfield et al. reported subtle but distinct changes in the salivary microbiome structure and composition in adults with depression compared to controls, noting significant differences in taxonomic abundance profiles (β-diversity) but no significant differences in α-diversity (1). These findings allowed for clear discrimination between healthy and depressed cohorts based on Bray–Curtis dissimilarities. Other studies, such as one by Simpson et al., focused on adolescents, suggesting that the composition, but not the diversity, of the oral microbiome is linked to anxiety and depression symptoms in a group of participants aged 14–18 (32, 44).

Despite varied conclusions in existing literature, these studies highlight the significant role of the oral microbiome in mental health, influenced potentially by limitation in sample size or the specific populations studied. Our research, utilizing data from the large cross-sectional NHANES study, observed significant associations between both α-diversity and β-diversity (using weighted unifrac distances) in structuring the oral microbiome and depression scores. The current study based on a large population-wide cohort allows for a more robust understanding of how oral microbiome diversity correlates with mental health outcomes across a general population. This study is among the first to use such a comprehensive dataset to explore the relationship between the oral microbiome and self-reported depression scores, enhancing our understanding of the microbiome’s impact on mental health.

The relationship between the oral microbiome and depression involves complex mechanisms. Researches have shown that specific microbial profiles in the oral cavity linked to increased inflammation and immune dysregulation, particularly in autoimmune diseases like rheumatoid arthritis and systemic lupus erythematosus, may contribute to depression (20, 45–48). Furthermore, studies comparing the microbial diversity in healthy individuals and those with autoimmune diseases highlight the critical role of a balanced oral microbiome in maintaining immune homeostasis. This evidence points to the need for further research to validate the mediating roles and establish the causal mechanisms of these inflammatory biomarkers in larger cohorts, enhancing our understanding of their impact on mental health and their potential as therapeutic targets.

While existing literature acknowledges the relationship between sleep and oral microbiome diversity with depression, understanding how the oral microbiome moderating the interaction between sleep and depression remains complex and underexplored. To date, research directly examining how the oral microbiome may regulate this relationship is still lacking. Our study delves into the role of oral microbial diversity in the relationship between sleep and depression, utilizing data from a large cross-sectional study NHANES. We showed that lower α-diversity within the lack of sleep category was associated with larger risk of depression, as evidenced by regression coefficients for various diversity metrics. For instance, lower OTU, Faith’s phylogenetic diversity, Shannon-Wiener diversity, and Inverse Simpson diversity in the lack of sleep group showed notable regression coefficients, indicating a more pronounced impact on depression. β-diversity metrics further elucidated the nuanced interaction between oral microbiome diversity and sleep duration on depression. Different clusters demonstrated varying effects, emphasizing the importance of considering the composition and structure of the oral microbiome. Notably, individuals with poor α-diversity in the oral microbiome and either insufficient or excessive sleep exhibited significantly increased depression scores compared to those with sufficient sleep.

In summary, our findings highlight the intricate interplay between sleep duration, depression, and oral microbiome diversity. Moreover, the moderating effects of oral microbiome diversity underscore the potential influence of oral health on the sleep-depression association. These insights contribute to a deeper understanding of the multifaceted factors contributing to mental health outcomes and may inform targeted interventions for individuals with specific sleep patterns and oral microbiome profiles.

One limitation of this study lies in the restricted access to summary data of raw oral microbial sequencing information, specifically limited to α-diversity and β-diversity metrics. The lack of detailed raw sequencing data hampers the depth of microbial analysis, impeding a comprehensive exploration of specific microbial taxa or functional elements. Moreover, the inability to annotate individual characteristics of cluster labels derived from the β-diversity index restricts the understanding of specific microbial compositions within each cluster, potentially overlooking valuable details that could enhance the depth and precision of the analysis and interpretation.

## Conclusion

Individuals exhibiting poor α-diversity in the oral microbiome alongside extreme sleep durations displayed notably higher depression scores compared to those achieving adequate sleep. These findings suggest a complex interplay between sleep patterns, oral microbiome diversity, and depression. However, it remains to be determined whether variations in oral microbiome diversity are directly influencing depression or merely reflect differences in oral health practices among those with depression. Further research is necessary to clarify these relationships and potentially inform more targeted mental health interventions.

## Data Availability

Publicly available datasets were analyzed in this study. This data can be found at: https://wwwn.cdc.gov/nchs/nhanes/Default.aspx.

## References

[1] Wingfield B Lapsley C McDowell A Miliotis G McLafferty M O’Neill SM. Variations in the oral microbiome are associated with depression in young adults. Sci Rep. (2021) 11:15009. doi: 10.1038/s41598-021-94498-6, PMID: 34294835 PMC8298414

[2] Liu QQ He H Yang J Feng X Zhao F Lyu J. Changes in the global burden of depression from 1990 to 2017: findings from the global burden of disease study. J Psychiatr Res. (2020) 126:134–40. doi: 10.1016/j.jpsychires.2019.08.002 31439359

[3] Sullivan PF Neale MC Kendler KS. Genetic epidemiology of major depression: review and meta-analysis. Am J Psychiatry. (2000) 157:1552–62. doi: 10.1176/appi.ajp.157.10.1552 11007705

[4] Nestler EJ Barrot M DiLeone RJ Eisch AJ Gold SJ Monteggia LM. Neurobiology of depression. Neuron. (2002) 34:13–25. doi: 10.1016/S0896-6273(02)00653-0 11931738

[5] Kessler RC. The effects of stressful life events on depression. Annu Rev Psychol. (1997) 48:191–214. doi: 10.1146/annurev.psych.48.1.191 9046559

[6] Vrshek-Schallhorn S Ditcheva M Corneau G. Stress in depression In: Harkness KL, and Hayden EP, editors. The Oxford Handbook of Stress and Mental Health. Oxford: Oxford University Press (2020). 97–126.

[7] Berding K Vlckova K Marx W Schellekens H Stanton C Clarke G. Diet and the microbiota-gut-brain Axis: sowing the seeds of good mental health. Adv Nutr. (2021) 12:1239–85. doi: 10.1093/advances/nmaa181 33693453 PMC8321864

[8] Shoubridge AP Choo JM Martin AM Keating DJ Wong ML Licinio J. The gut microbiome and mental health: advances in research and emerging priorities. Mol Psychiatry. (2022) 27:1908–19. doi: 10.1038/s41380-022-01479-w 35236957

[9] Halverson T Alagiakrishnan K. Gut microbes in neurocognitive and mental health disorders. Ann Med. (2020) 52:423–43. doi: 10.1080/07853890.2020.1808239 32772900 PMC7877977

[10] Cryan JF Dinan TG. Mind-altering microorganisms: the impact of the gut microbiota on brain and behaviour. Nat Rev Neurosci. (2012) 13:701–12. doi: 10.1038/nrn3346 22968153

[11] Järbrink-Sehgal E Andreasson A. The gut microbiota and mental health in adults. Curr Opin Neurobiol. (2020) 62:102–14. doi: 10.1016/j.conb.2020.01.016 32163822

[12] Gao L Xu T Huang G Jiang S Gu Y Chen F. Oral microbiomes: more and more importance in oral cavity and whole body. Protein Cell. (2018) 9:488–500. doi: 10.1007/s13238-018-0548-1, PMID: 29736705 PMC5960472

[13] Agnello M Marques J Cen L Mittermuller B Huang A Chaichanasakul Tran N. Microbiome associated with severe caries in Canadian first nations children. J Dent Res. (2017) 96:1378–85. doi: 10.1177/0022034517718819, PMID: 28709393 PMC5652857

[14] Kaan AM Kahharova D Zaura E. Acquisition and establishment of the oral microbiota. Periodontology. (2000) 86:123–41. doi: 10.1111/prd.12366 PMC8252790 33690935

[15] Thomas C Minty M Vinel A Canceill T Loubières P Burcelin R. Oral microbiota: a major player in the diagnosis of systemic diseases. Diagnostics. (2021) 11:1376. doi: 10.3390/diagnostics11081376, PMID: 34441309 PMC8391932

[16] Kumar PS. From focal sepsis to periodontal medicine: a century of exploring the role of the oral microbiome in systemic disease. J. Physiol. (2017) 595:465–76. doi: 10.1113/JP272427, PMID: 27426277 PMC5233655

[17] Hou K Wu ZX Chen XY Wang JQ Zhang D Xiao C. Microbiota in health and diseases. Signal Transduct Target Ther. (2022) 7:135. doi: 10.1038/s41392-022-00974-4, PMID: 35461318 PMC9034083

[18] Tonelli A Lumngwena EN Ntusi NAB. The oral microbiome in the pathophysiology of cardiovascular disease. Nat Rev Cardiol. (2023) 20:386–403. doi: 10.1038/s41569-022-00825-3 36624275

[19] Long J Cai Q Steinwandel M Hargreaves MK Bordenstein SR Blot WJ. Association of oral microbiome with type 2 diabetes risk. J Periodontal Res. (2017) 52:636–43. doi: 10.1111/jre.12432, PMID: 28177125 PMC5403709

[20] Zhang X Zhang D Jia H Feng Q Wang D Liang D. The oral and gut microbiomes are perturbed in rheumatoid arthritis and partly normalized after treatment. Nat Med. (2015) 21:895–905. doi: 10.1038/nm.3914, PMID: 26214836

[21] Richardson BN Noh HI Webster CI Zhang W Kim S Yang I. Oral microbiome, mental health, and sleep outcomes during the COVID-19 pandemic: an observational study in Chinese and Korean American immigrants. Omics. (2023) 27:180–90. doi: 10.1089/omi.2022.0182, PMID: 36946910 PMC10122216

[22] Botelho J Mascarenhas P Viana J Proença L Orlandi M Leira Y. An umbrella review of the evidence linking oral health and systemic noncommunicable diseases. Nat Commun. (2022) 13:7614. doi: 10.1038/s41467-022-35337-8, PMID: 36494387 PMC9734115

[23] Kunath BJ Hickl O Queirós P Martin-Gallausiaux C Lebrun LA Halder R. Alterations of oral microbiota and impact on the gut microbiome in type 1 diabetes mellitus revealed by integrated multi-omic analyses. Microbiome. (2022) 10:243. doi: 10.1186/s40168-022-01435-4, PMID: 36578059 PMC9795701

[24] Wade WG. The oral microbiome in health and disease. Pharmacol Res. (2013) 69:137–43. doi: 10.1016/j.phrs.2012.11.006 23201354

[25] Dinan TG Cryan JF. Brain-gut-microbiota axis and mental health. Psychosom Med. (2017) 79:920–6. doi: 10.1097/PSY.0000000000000519 28806201

[26] Vogtmann E Chaturvedi AK Blaser MJ Bokulich NA Caporaso JG Gillison ML. Representative oral microbiome data for the US population: the National Health and nutrition examination survey. Lancet Microbe. (2023) 4:E60–1. doi: 10.1016/S2666-5247(22)00333-0 36455567

[27] Chaput JP Dutil C Featherstone R Ross R Giangregorio L Saunders TJ. Sleep timing, sleep consistency, and health in adults: a systematic review. Appl Physiol Nutr Metab. (2020) 45:S232–47. doi: 10.1139/apnm-2020-0032, PMID: 33054339

[28] Seweryn P Orzeszek SM Waliszewska-Prosół M Jenča A Osiewicz M Paradowska-Stolarz A. Relationship between pain severity, satisfaction with life and the quality of sleep in polish adults with temporomandibular disorders. Dent Med Probl. (2023) 60:609–17. doi: 10.17219/dmp/171894 37873974

[29] Mason GM Lokhandwala S Riggins T Spencer RMC. Sleep and human cognitive development. Sleep Med Rev. (2021) 57:101472. doi: 10.1016/j.smrv.2021.101472, PMID: 33827030 PMC8164994

[30] Pandi-Perumal SR Monti JM Burman D Karthikeyan R BaHammam AS Spence DW. Clarifying the role of sleep in depression: a narrative review. Psychiatry Res. (2020) 291:113239. doi: 10.1016/j.psychres.2020.113239, PMID: 32593854

[31] Scott AJ Webb TL Martyn-St James M Rowse G Weich S. Improving sleep quality leads to better mental health: a meta-analysis of randomised controlled trials. Sleep Med Rev. (2021) 60:101556. doi: 10.1016/j.smrv.2021.101556, PMID: 34607184 PMC8651630

[32] Li J Cao D Huang Y Wang R Dong Q Wei Q. Sleep duration and health outcomes: an umbrella review. Sleep Breath. (2022) 26:1479–501. doi: 10.1007/s11325-021-02458-1 34435311

[33] Zhai L Zhang H Zhang D. Sleep duration and depression among adults: a meta-analysis of prospective studies. Depress Anxiety. (2015) 32:664–70. doi: 10.1002/da.22386 26047492

[34] Watson NF Badr MS Belenky G Bliwise DL Buxton OM Buysse D. Recommended amount of sleep for a healthy adult: a joint consensus statement of the American Academy of sleep medicine and Sleep Research Society. J Clin Sleep Med. (2015) 11:591–2. doi: 10.5664/jcsm.4758, PMID: 25979105 PMC4442216

[35] Shivappa N Steck SE Hurley TG Hussey JR Hébert JR. Designing and developing a literature-derived, population-based dietary inflammatory index. Public Health Nutr. (2014) 17:1689–96. doi: 10.1017/S1368980013002115, PMID: 23941862 PMC3925198

[36] Hirshkowitz M Whiton K Albert SM Alessi C Bruni O DonCarlos L. National Sleep Foundation's sleep time duration recommendations: methodology and results summary. Sleep Health. (2015) 1:40–3. doi: 10.1016/j.sleh.2014.12.010, PMID: 29073412

[37] Manea L Gilbody S McMillan D. A diagnostic meta-analysis of the patient health Questionnaire-9 (PHQ-9) algorithm scoring method as a screen for depression. Gen Hosp Psychiatry. (2015) 37:67–75. doi: 10.1016/j.genhosppsych.2014.09.009 25439733

[38] Ingwer Borg PJFG. Modern multidimensional scaling: Theory and applications. Berlin: Springer Science & Business Media (2005).

[39] Hornig M. The role of microbes and autoimmunity in the pathogenesis of neuropsychiatric illness. Curr Opin Rheumatol. (2013) 25:488–795. doi: 10.1097/BOR.0b013e32836208de, PMID: 23656715

[40] Larsen JM. The immune response to Prevotella bacteria in chronic inflammatory disease[J]. Immunology. (2017) 151:363–74. doi: 10.1111/imm.12760, PMID: 28542929 PMC5506432

[41] Peruzzo DC Benatti BB Ambrosano GMB Nogueira-Filho GR Sallum EA Casati MZ. A systematic review of stress and psychological factors as possible risk factors for periodontal disease. J Periodontol. (2007) 78:1491–504. doi: 10.1902/jop.2007.060371, PMID: 17668968

[42] Eyre HA Lavretsky H Kartika J Qassim A Baune BT. Modulatory effects of antidepressant classes on the innate and adaptive immune system in depression. Pharmacopsychiatry. (2016) 49:85–96. doi: 10.1055/s-0042-103159 26951496 PMC5548302

[43] Baglioni C Battagliese G Feige B Spiegelhalder K Nissen C Voderholzer U. Insomnia as a predictor of depression: a meta-analytic evaluation of longitudinal epidemiological studies. J Affect Disord. (2011) 135:10–9. doi: 10.1016/j.jad.2011.01.011, PMID: 21300408

[44] Simpson CA Adler C du Plessis MR Landau ER Dashper SG Reynolds EC. Oral microbiome composition, but not diversity, is associated with adolescent anxiety and depression symptoms. Physiol Behav. (2020) 226:113126. doi: 10.1016/j.physbeh.2020.113126 32777312

[45] Han YW Wang X. Mobile microbiome: oral bacteria in extra-oral infections and inflammation. J Dent Res. (2013) 92:485–91. doi: 10.1177/0022034513487559, PMID: 23625375 PMC3654760

[46] Said HS Suda W Nakagome S Chinen H Oshima K Kim S. Dysbiosis of salivary microbiota in inflammatory bowel disease and its association with oral immunological biomarkers. DNA Res. (2014) 21:15–25. doi: 10.1093/dnares/dst037, PMID: 24013298 PMC3925391

[47] Kohn JN Kosciolek T Marotz C Aleti G Guay-Ross RN Hong SH. Differing salivary microbiome diversity, community and diurnal rhythmicity in association with affective state and peripheral inflammation in adults. Brain Behav Immun. (2020) 87:591–602. doi: 10.1016/j.bbi.2020.02.004 32061904 PMC10287023

[48] Paradowska-Gorycka A Wajda A. How the gut microbiota contributes to changes of autoimmune phenotype–from molecular studies to clinical utility. Reumatologia. (2020) 58:189–90. doi: 10.5114/reum.2020.98427 32921823 PMC7477474

