# Supplementary material for: Oral microbiome diversity shapes the association between sleep duration and depression
Source: Front Neurol. 2024 Sep 13;15:1442557. doi: 10.3389/fneur.2024.1442557 (PMC11427320; doi:10.3389/fneur.2024.1442557)
Supplement: Supplementary file 1 [file Data_Sheet_1.docx]

**Supplementary Materials**

**
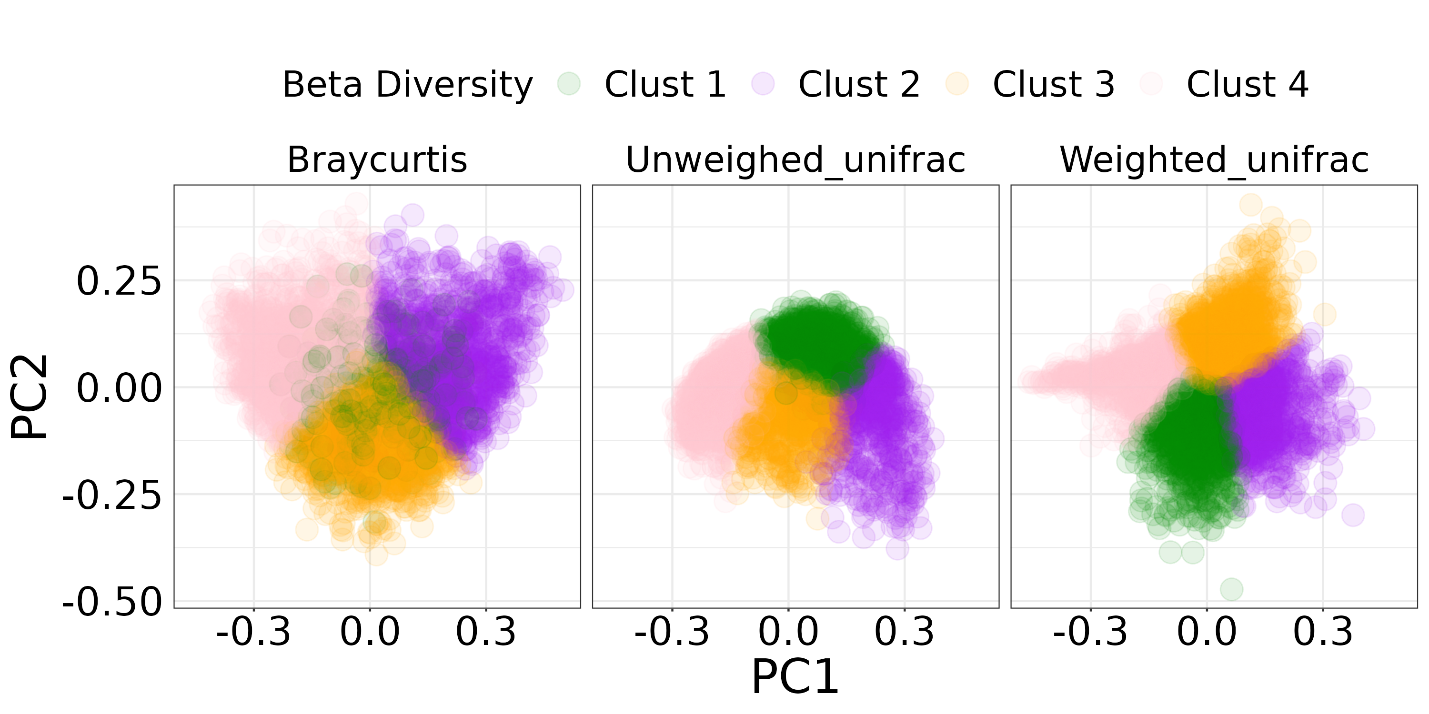
Figure S1.** Visualization of samples organized into four clusters based on labels derived from k-means clustering using β-diversity metrics (Bray-Curtis, unweighted, and weighted UniFrac distances). The number of clusters was established using the silhouette criterion.
